# Feature-tracking cardiac magnetic resonance left ventricular global longitudinal strain improves 6 months after kidney transplantation associated with reverse remodeling, not myocardial tissue characteristics

**DOI:** 10.1007/s10554-021-02284-2

**Published:** 2021-05-17

**Authors:** Maurício Fregonesi Barbosa, Mariana Moraes Contti, Luis Gustavo Modelli de Andrade, Alejandra del Carmen Villanueva Mauricio, Sergio Marrone Ribeiro, Gilberto Szarf

**Affiliations:** 1 Department of Diagnostic Imaging, Escola Paulista de Medicina, Universidade Federal de São Paulo (UNIFESP), Rua Napoleão de Barros 800, Vila Clementino, São Paulo, Brazil; 2 Nephrology Division, Department of Internal Medicine, Universidade Estadual Paulista (UNESP), Botucatu, Brazil; 3 Cardiology Division, Department of Internal Medicine, Universidade Estadual Paulista (UNESP), Botucatu, Brazil; 4 Department of Tropical Diseases and Diagnostic Imaging, Universidade Estadual Paulista (UNESP), Botucatu, Brazil; 5 Hospital Israelita Albert Einstein, São Paulo, Brazil

**Keywords:** Strain, Cardiovascular magnetic resonance imaging, Native T1, Subclinical cardiac dysfunction, Renal transplant

## Abstract

To determine whether left ventricular (LV) global longitudinal strain (GLS) measured by feature-tracking (FT) cardiac magnetic resonance (CMR) improves after kidney transplantation (KT) and to analyze associations between LV GLS, reverse remodeling and myocardial tissue characteristics. This is a prospective single-center cohort study of kidney transplant recipients who underwent two CMR examinations in a 3T scanner, including cines, tagging, T1 and T2 mapping. The baseline exam was done up to 10 days after transplantation and the follow-up after 6 months. Age and sex-matched healthy controls were also studied for comparison. A total of 44 patients [mean age 50 ± 11 years-old, 27 (61.4%) male] completed the two CMR exams. LV GLS improved from − 13.4% ± 3.0 at baseline to − 15.2% ± 2.7 at follow-up (p < 0.001), but remained impaired when compared with controls (− 17.7% ± 1.5, p = 0.007). We observed significant correlation between improvement in LV GLS with reductions of left ventricular mass index (r = 0.356, p = 0.018). Improvement in LV GLS paralleled improvements in LV stroke volume index (r = − 0.429, p = 0.004), ejection fraction (r = − 0.408, p = 0.006), global circumferential strain (r = 0.420, p = 0.004) and global radial strain (r = − 0.530, p = 0.002). There were no significant correlations between LV GLS, native T1 or T2 measurements (p > 0.05). In this study, we demonstrated that LV GLS measured by FT-CMR improves 6 months after KT in association with reverse remodeling, but not native T1 or T2 measurements.

## Introduction

Cardiovascular disease (CVD) remains the leading cause of death in patients with chronic kidney disease (CKD) and end-stage renal disease (ESRD) [1]. This increased cardiovascular risk is mainly related to changes in cardiac structure and function named uremic cardiomyopathy (UC) [2, 3]. The histologic basis for UC is cardiomyocyte hypertrophy and increased interstitial myocardial fibrosis [4, 5], that eventually causes myocardial dysfunction. Kidney transplantation (KT) is considered the most effective form of ESRD treatment and is associated with reverse remodeling, improved ventricular function and better outcomes [6], however kidney transplant recipients (KTR) are still at increased cardiovascular risk compared to the general population [7, 8].

Cardiac magnetic resonance (CMR) is the gold standard to measure cardiac structure and function, having the further advantage to non-invasively characterize myocardial tissue using T1 and T2 mapping [9], without intravenous gadolinium injection, which is of great interest in renal insufficiency because of the risk of nephrogenic systemic fibrosis (NSF) [10]. The recent development of feature-tracking techniques (FT-CMR) now allow assessment of global longitudinal strain (GLS) from standard cine images without the need for other specialized pulse sequences or additional scanning time [11]. Previous CMR studies have shown subclinical features of myocardial disease characterized by reduced left ventricular (LV) GLS and increased myocardial fibrosis, as assessed by T1 mapping, in CKD [12] and ESRD [13, 14]. Although KT was associated with reduced myocardial fibrosis [15], previous FT-CMR [16] and speckle-tracking echocardiography (STE) [17, 18] studies found different results about the effects of KT in LV GLS, which is the most reliable and studied strain parameter. Nevertheless, the relationships between LV GLS, cardiac structure and myocardial tissue characteristics in this setting are unknown. Accordingly, in this study we sought to evaluate whether LV GLS measured by FT-CMR improves after KT and analyze associations between LV GLS, cardiac structure (mass and volumes) and myocardial tissue characteristics (native T1 and T2). In addition, we compared CMR data of KTR at follow-up with healthy volunteers to analyze whether KT could reverse these biomarkers of UC.

## Methods

### Study design and participants

This is a single-center prospective cohort study in a university hospital with ESRD patients who received a kidney transplant and underwent two CMR examinations. The first exam (baseline) was performed between the 1st and the 10th postoperative days. The second one was performed 6 months after renal transplantation. We included consecutive patients over 18 years-old who received a kidney transplant from a living or deceased donor. We excluded patients with a contraindication to CMR (e.g., pacemaker, cochlear implant, cerebral aneurysm clip, tattooing, claustrophobia) or inability to perform breath hold. For comparison a group of age- and sex-matched healthy controls was selected from the hospital records. Healthy subjects had normal kidney function, no known chronic disease, and were not on regular medication.

This study complied with the Declaration of Helsinki, and the institutional review board of Botucatu Medical School-UNESP approved the research protocol (approval number: 972.129). All participants provided witnessed, written, informed consent. Siemens Healthineers (Erlangen, Germany) provided the use of work-in-progress #448B (VB17A) quantitative cardiac parameter mapping (T1|T2|T2*) in this study. No person from this company had access to study data or was involved in image analysis, manuscript preparation, or any part of the study. The authors had full control of the data submitted for publication.

### CMR technique and measurements

All patients underwent their examination on a 3.0-T Magnetom Verio Scanner (Siemens, Erlangen, Germany) with a phased array chest coil, according to study protocol. A cardiac cine balanced steady-state free precession (bSSFP) sequence was acquired using retrospective cardiac gating. Typically, 25 phases were acquired in 2-, 3-, and 4-chamber long axis views and a stack of short axis views. Scan parameters: field of view (FOV) 37 cm, repetition time (TR) 43.54 ms, echo time (TE) 1.38 ms, flip angle (FA) 50°, slice thickness 6 mm, in-plane image resolution 1.6 × 1.6 mm. Myocardial tissue tagging was performed with an ECG-gated line tagging sequence with complementary spatial modulation of magnetization (CSPAMM). Image parameters were: FOV 32 cm, TR 48.15, TE 2.54, FA 10°, slice thickness 7 mm with a tag spacing of 7 mm. Short-axis tissue tagging was performed on three levels of the LV, positioned at 25%, 50% and 75% of the distance between the mitral valve annulus and the apex on a LV 4-chamber view in end-systole, and in 2- and 4-chamber long axis views. Quantitative T2 mapping was performed using a T2-prepared SSFP sequence in a mid-ventricular short axis view with the following imaging parameters: FOV 36 cm, TR 254.32, TE 1.07 ms, flip angle 35°, slice thickness 8 mm, in-plane image resolution 2.5 × 1.9 mm, acquisition in late diastole on every fourth heartbeat; T2 preparations: 0 ms, 25 ms and 55 ms. Quantitative T1 mapping was performed with a Modified Look-Locker Inversion-Recovery (MOLLI) sequence in mid-ventricular short axis view without intravenous contrast injection (Native T1). Scan parameters: FOV 36 cm, TR 316.09, TE 1.12 ms, flip angle 35°, slice thickness 8 mm, in-plane image resolution 2.1 × 1.4 mm, acquisition in late diastole on every other heartbeat, minimal inversion time 120 ms; increment 80 ms. The T1 mapping scheme included 5 acquisitions after the first inversion pulse, followed by a 3-heartbeat pause and a second inversion pulse followed by three acquisitions [5(3)3].

### CMR analysis

The biventricular end-diastolic volume (EDV) and end-systolic volume (ESV) were measured by manual segmentation of the short axis cine images, using Argus function software (Siemens, Erlangen, Germany). The endocardial borders were traced at end-diastole and end-systole, including trabeculations and papillary muscles in the blood pool. EDV and ESV were calculated for each ventricle using the disc summation method. Ventricular stroke volume (SV) was calculated as the difference between the EDV and ESV, and ventricular ejection fraction (EF) was (SV/EDV) × 100. LV epicardial borders were drawn only at end-diastole to calculate LV mass (LVM). All volume measurements were indexed for the body surface area (BSA) and expressed in ml/m^2^.

**Fig. 1:**
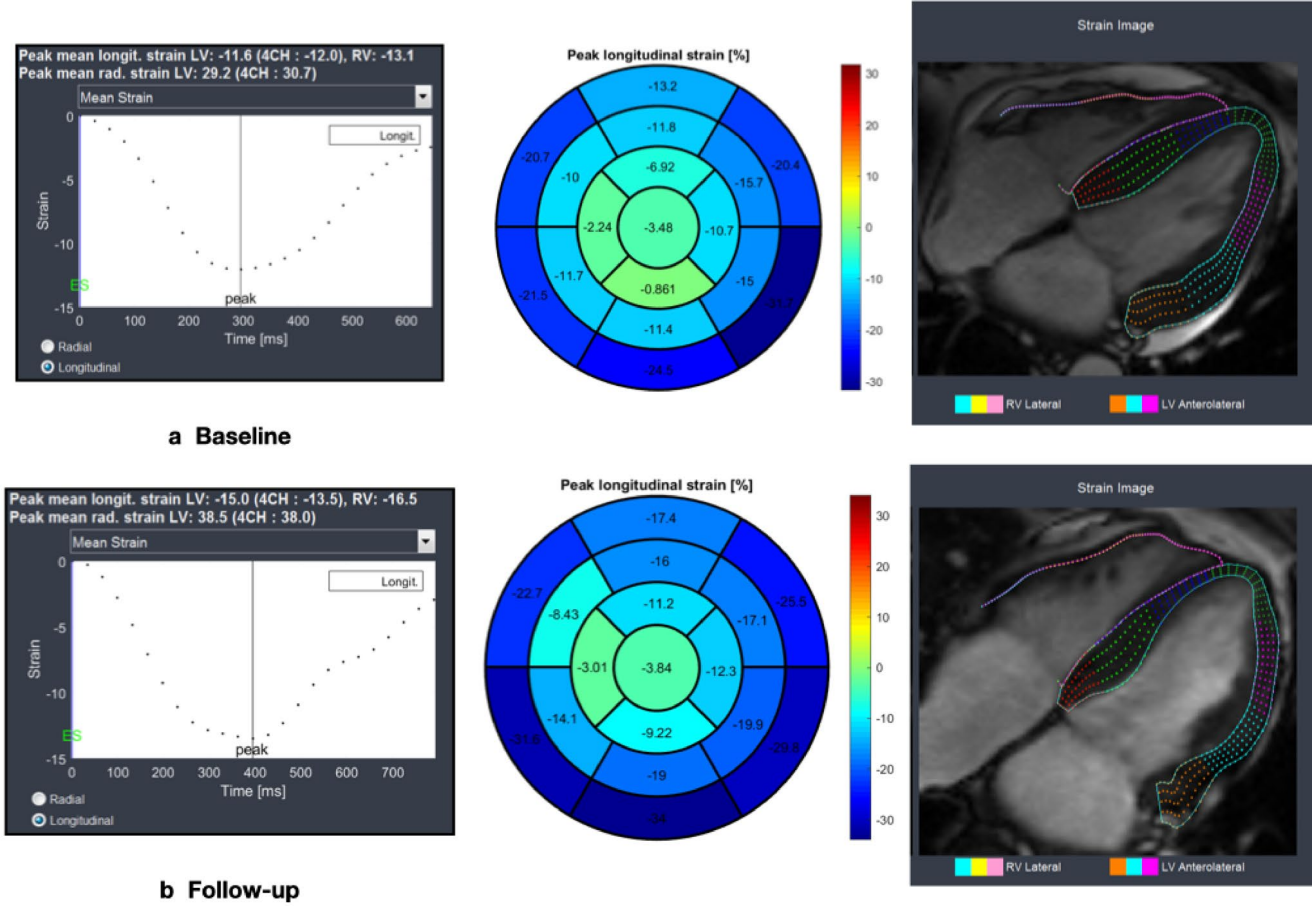
Example of global longitudinal strain by FT-CMR in a 52-year-old man, living-donor kidney transplant patient. Top: Baseline (LV GLS = − 11.6% and RV GLS = − 13.1%), Bottom: Follow-up (LV GLS = − 15.0% and RV GLS = − 16.5%)

Myocardial feature-tracking analysis was performed processing cine images using strain module of Segment Medviso software, which was previously validated in a clinical setting [19]. Circumferential and radial strains were analyzed in basal, medial and apical short axis slices by manual segmentation of the LV blood pool cavity and myocardium, while longitudinal strains were analyzed in 2-, 3- and 4-chamber long axis views. This last long axis view was also used for RV analysis after manual segmentation of RV endocardial borders. Strain values were obtained for each segment and global values defined as the mean of all segmental values. For validation, tagging strain analysis was performed using the same software to process tagged long axis views. Figure 1 displays an example of GLS feature-tracking analysis at baseline and follow-up CMR exams.

T1 and T2 maps were automatically generated on the MR scanner with motion corrected images using a novel non-rigid registration algorithm [20, 21]. A region of interest (ROI) was then drawn conservatively in the septal myocardium for each map, according to previous consensus [22].

An experienced reader (ACVM) measured ventricular volumes, mass and EF, while another experienced reader (MFB) independently performed T1, T2 and strain analysis, blinded to former results.

### Analysis of reproducibility and validation of LV GLS measurements

To determine the intraobserver reproducibility of LV GLS measured by FT-CMR, 15 exams were randomly selected and the analysis repeated by the same observer about 6 months after the initial assessment. These exams were also used to validate LV GLS measured by FT-CMR against the reference standard tagging.

### Statistical analysis

The Kolmogorov–Smirnov test was applied to determine appropriate parametric or nonparametric tests. Quantitative variables were expressed as mean ± standard deviation or median (interquartile range) and compared by t test or Wilcoxon signed-rank test, whereas qualitative variables were expressed by their frequencies and percentages, and compared by the chi-square test or Fisher’s exact test. The relationship between changes in LV GLS and variables of interest were assessed using Pearson’s correlation coefficients for continuous normally distributed variables and Spearman’s correlation for categorical or non-normally distributed data. Linear regression analysis was used to evaluate the influence of clinical variables in LV GLS changes. Intraobserver reproducibility was assessed by analyzing Bland–Altman plot. All data were analyzed using SAS Studio 3.8 or Microsoft Excel software. A p value of ≤ 0.05 was considered significant.

## Results

### Participants

We consented 47 patients of whom 44 [Mean age 50 ± 11 years-old; 27 (61.4%) male] completed the two CMR exams (n = 88 CMR studies). One patient died on the 11th day after transplant surgery and two patients refused to undergo a second CMR exam. The median time from transplant to the first exam (baseline) was 5 days and to the second exam (follow-up) was 189 days. During the study period, no patient developed a graft loss or a cardiovascular event (acute myocardial infarction, acute coronary syndrome or arrhythmias). Control group was composed by 10 age- and sex-matched healthy controls selected from the hospital records. Table 1 describes the subject characteristics for KT (baseline and follow-up) and control groups.

**Table 1:** Clinical characteristics of kidney transplant patients (baseline and follow-up) and healthy controls

	Baseline (n = 44)	Follow-up (n = 44)	Controls (n = 10)
Age (years)	50 ± 11	n/a	48 ± 13
Male	27 (61,4%)	n/a	6 (60,0%)
Dialysis vintage (months)	29 [15–45]	n/a	n/a
Cause of ESRD			
Hypertension	11 (25,0%)	n/a	n/a
Diabetes	11 (25,0%)	n/a	n/a
Glomerulonephritis	8 (18,2%)	n/a	n/a
Unknown	7 (15,9%)	n/a	n/a
Others	7 (15,9%)	n/a	n/a
BMI (kg/m^2^)	25 ± 05	26 ± 04	26 ± 03
HR (bpm)	86 ± 13	79 ± 12*	57 ± 05**
DBP (mmHg)	80 [70–90]	70 [70–80]*	80 [80–80]
SBP (mmHg)	135 [130–150]	120 [110–140]*	120 [120–120]
Creatinine (mg/dL)	4.2 [2–7.7]	1.3 [1–1.8]*	0.8 [0.7–0.9]**
Ht (%)	32.7 ± 5.9	38.4 ± 4.9*	44.6 ± 3.7**

Continuous variables expressed as mean and standard deviation (mean ± standard deviation) or median and 25 and 75% percentiles [median (25th and 75th percentile)]

*n/a* Not applicable, *BMI* body mass index, *DBP* diastolic blood pressure, *ESRD* end-stage renal disease, *HR* heart rate, *Ht* hematocrit, *SBP* systolic blood pressure

*Indicates p value < 0.05 between paired data at baseline and follow-up

**Indicates p value < 0.05 between unpaired data comparing the transplant cohort at follow-up and healthy controls

### CMR parameters

**Fig. 2:**
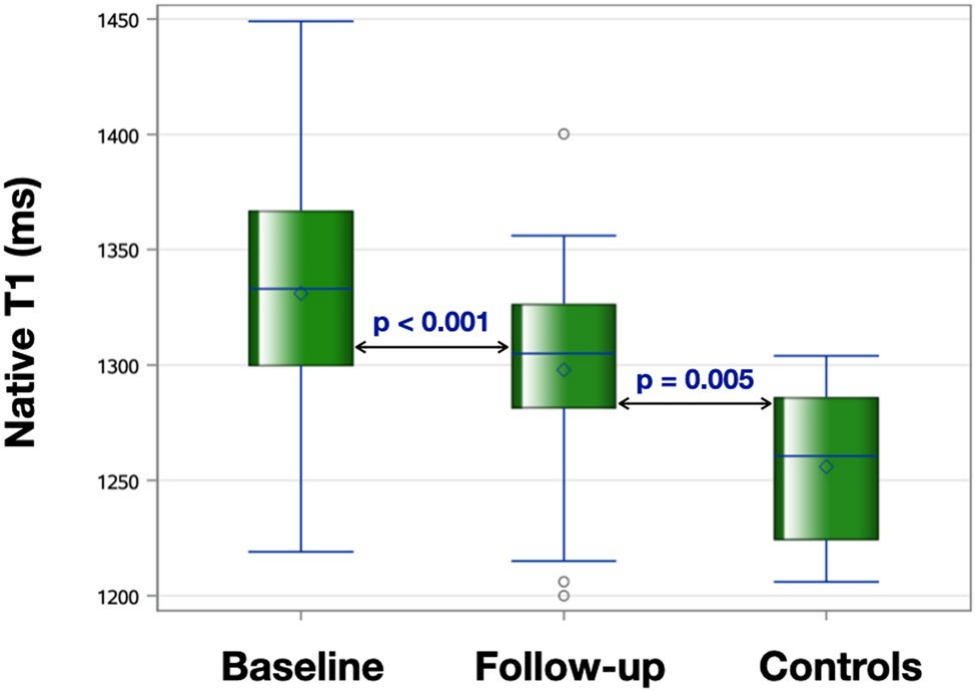
Boxplots comparing Native T1 at baseline and 6 months after transplantation with controls

There were no significant changes in LV volumes, mass or EF. The mean native T1 decreased from 1331 ± 52 to 1298 ± 42 ms at 6 months (p < 0.001), but still remained higher than controls (1256 ± 33 ms, p = 0.005) (Fig. 2). There was no change in T2 times, suggesting that reduction in native T1 was probably related to regression of myocardial fibrosis, not edema. Also, there were no significant changes in right ventricular (RV) volumes or EF. Table 2 summarizes CMR variables for KT (baseline and follow-up) and control groups.

**Table 2:** Cardiac magnetic resonance variables for KT patients (baseline and follow-up) and controls

	Baseline (n = 44)	Follow-up (n = 44)	Controls (n = 10)
LV EF (%)	64 ± 12	67 ± 10	68 ± 03
LV EDVi (ml/m^2^)	88 ± 23	85 ± 19	76 ± 16
LV ESVi (ml/m^2^)	30 [21–40]	28 [22–36]	20 [20-27]
LV SVi (ml/m^2^)	55 ± 12	56 ± 11	54 ± 10
LV Mi (g/m^2^)	87 ± 20	85 ± 16	67 ± 11**
Native T1 (ms)	1331 ± 52	1298 ± 42*	1256 ± 33**
T2 (ms)	43 ± 04	43 ± 03	41 ± 01**
RV EF (%)	65 ± 11	62 ± 07	67 ± 07**
RV EDVi (ml/m^2^)	71 ± 20	74 ± 15	79 ± 14
RV ESVi (ml/m^2^)	26 ± 14	28 ± 9	26 ± 9
RV SVi (ml/m^2^)	45 ± 14	46 ± 11	53 ± 08

Continuous variables expressed as mean and standard deviation (mean ± standard deviation) or median and 25 and 75% percentiles [median (25th and 75th percentile)]

*EDVi* End-diastolic volume index, *EF* ejection fraction, *ESVi* end-systolic volume index, *LV* left ventricular, *Mi* mass index, *RV* right ventricular, *SVi* stroke volume index

*Indicates p value < 0.05 between paired data at baseline and follow-up

**Indicates p value < 0.05 between unpaired data comparing the transplant cohort at follow-up and healthy controls

### Strain by FT-CMR

**Fig. 3:**
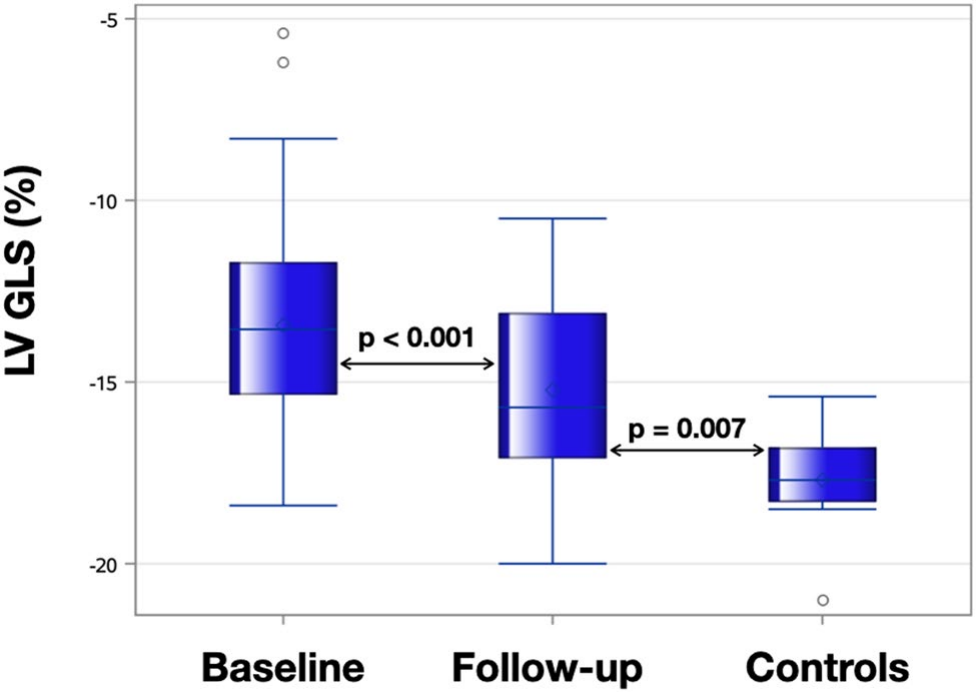
Boxplots comparing left ventricular global longitudinal strain (LV GLS) at baseline and 6 months after transplantation with controls

**Fig. 4:**
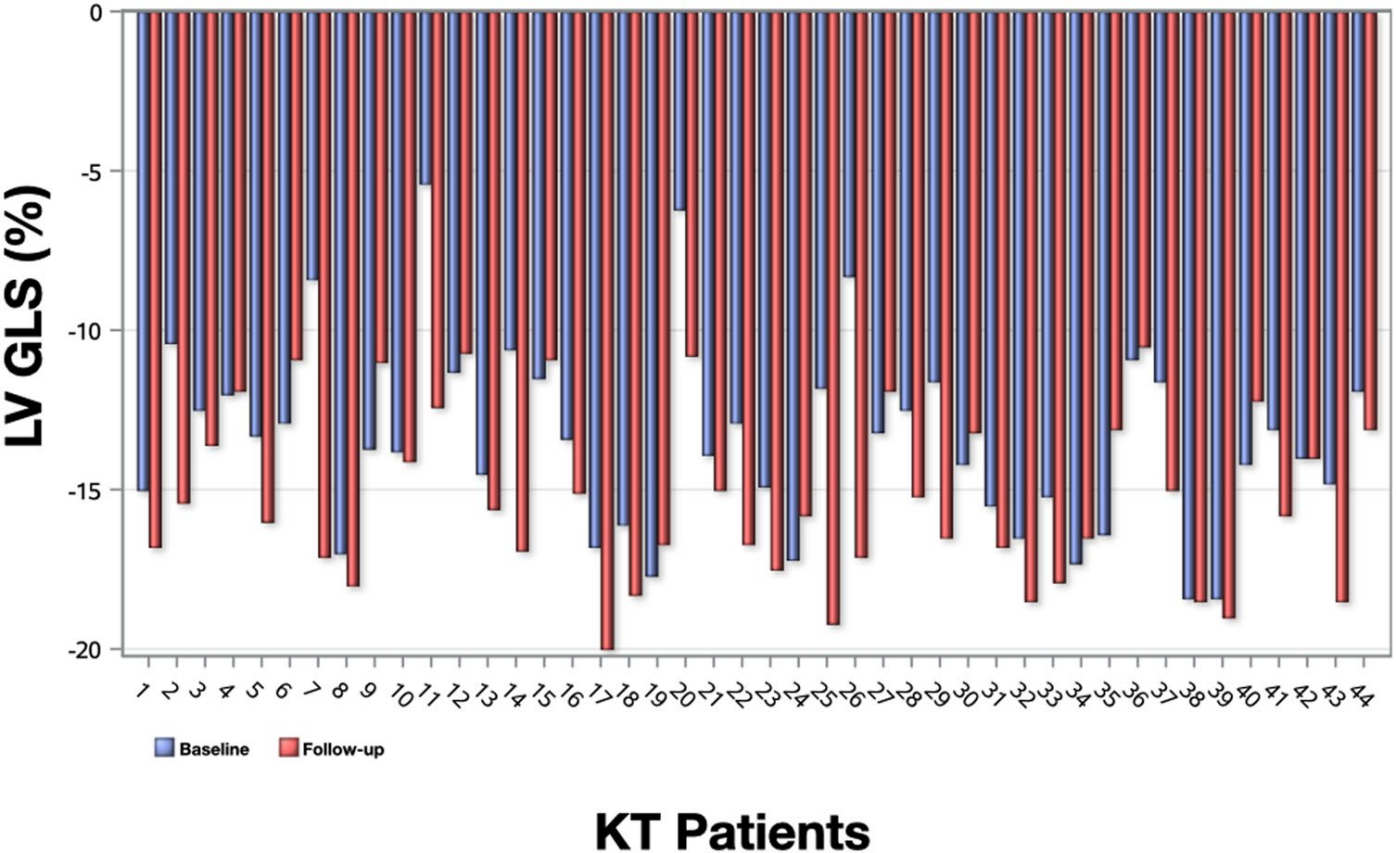
Analysis of individual cases of left ventricular global longitudinal strain (LV GLS) in kidney transplant (KT) patients at baseline and follow-up (n = 44)

Compared to baseline LV GLS improved from − 13.4% ± 3.0 to − 15.2% ± 2.7 (p < 0.001), LV basal global circumferential strain (GCS) improved from − 16.7% ± 3.5 to − 18.2% ± 2.0 (p = 0.002) and RV GLS improved from − 11.5% ± 3.9 to − 14.1% ± 4.1 (p < 0.001) after 6 months of KT. The other strain variables remained unchanged. Besides these improvements, LV GLS and RV GLS remained impaired at follow-up when compared to controls [− 15.2% ± 2.7 versus − 17.7% ± 1.5, p = 0.007 (Fig. 3) and − 14.1% ± 4.1 versus − 18.0% ± 2.4, p = 0.005, respectively]. The analysis of individual cases demonstrated that the majority of the patients had improvements in LV GLS values between baseline and 6 months after transplantation (Fig. 4). Table 3 summarizes strain measurements by FT-CMR for KT (baseline and follow-up) and control groups.

**Table 3:** Strain by feature-tracking CMR in KT patients (baseline and follow-up) and controls

	Baseline (n = 44)	Follow-up (n = 44)	Controls (n = 10)
Basal GCS (%)	− 16.7 ± 3.5	− 18.2 ± 2.8*	n/a
Medio GCS (%)	− 17.2 ± 4.2	− 17.7 ± 3.6	n/a
Apical GCS (%)	− 20.1 ± 5.2	− 20.3 ± 5.1	n/a
LV GCS (%)	− 18.0 ± 4.0	− 18.7 ± 3.3	− 19.9 ± 2.3
Basal GRS (%)	36.1 ± 14.4	37.7 ± 12.6	n/a
Medio GRS (%)	48.4 ± 16.8	49.6 ± 14.4	n/a
Apical GRS (%)	49.2 ± 15.1	51.3 ± 17.0	n/a
LV GRS (%)	44.6 ± 13.8	46.2 ± 12.6	41.3 ± 8.9
2CH GLS (%)	− 13.9 ± 3.4	− 16.0 ± 3.7	n/a
4CH GLS (%)	− 12.8 ± 3.1	− 14.4 ± 3.1	n/a
3CH GLS (%)	− 13.6 ± 3.2	− 15.2 ± 3.4	n/a
LV GLS (%)	− 13.4 ± 3.0	− 15.2 ± 2.7*	− 17.7 ± 1.5**
RV GLS (%)	− 11.5 ± 3.9	− 14.1 ± 4.1*	− 18.0 ± 2.4**

Data expressed as mean and standard deviation (mean ± standard deviation)

n/a Not applicable, *CH* chamber, *GCS* global circumferential strain, *GLS* global longitudinal strain, *GRS* global radial strain, *LV* left ventricular, *RV* right ventricular

*Indicates p value < 0.05 between paired data at baseline and transplant follow-up

**Indicates p value < 0.05 between unpaired data comparing the transplant cohort at follow-up and healthy controls

### Association between LV GLS, CMR and clinical variables

**Fig. 5:**
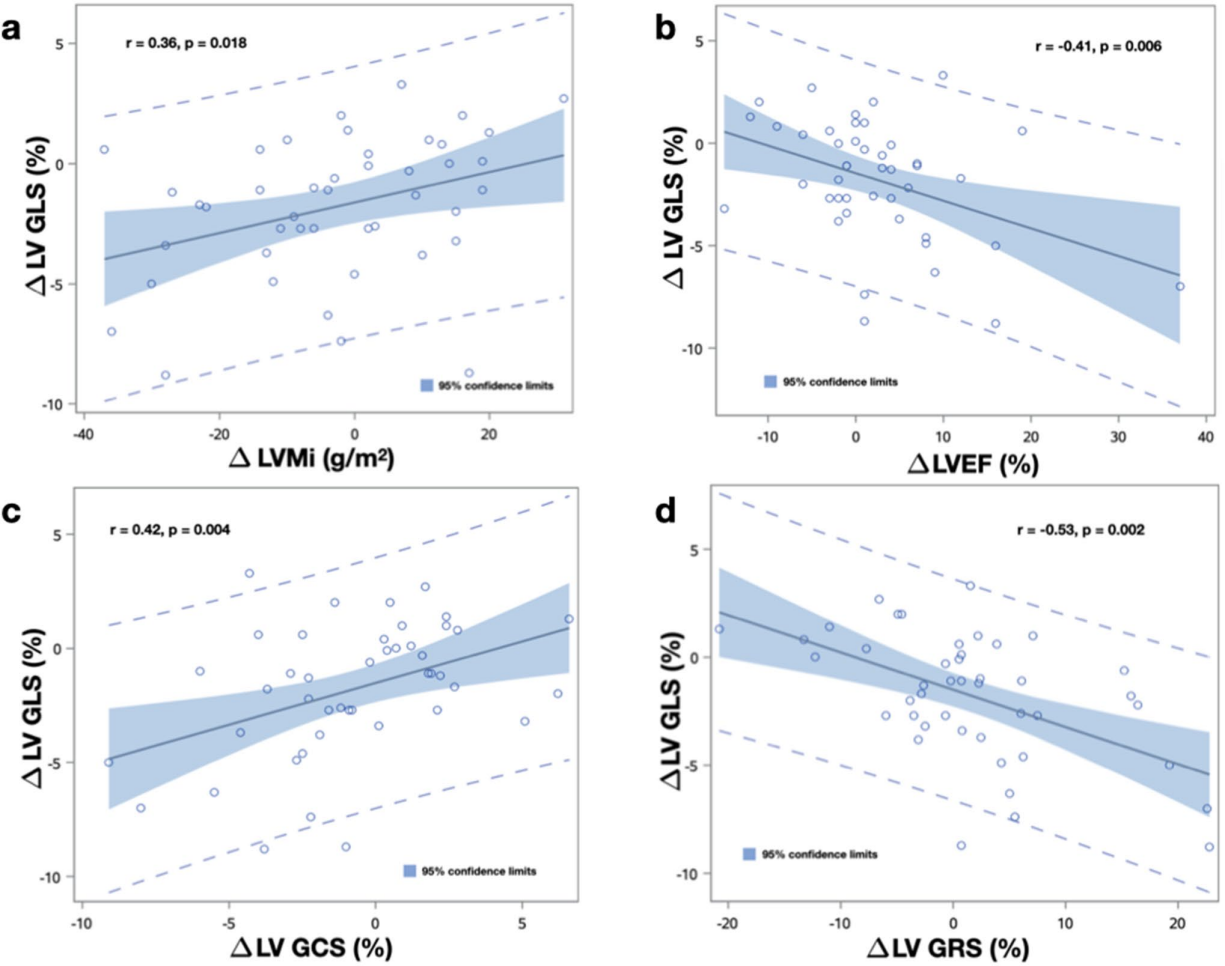
Relationship between changes in left ventricular global longitudinal strain (∆ LV GLS) with changes in **a** mass index (∆ LVMi), **b** ejection fraction (∆ LVEF), **c** global circumferential strain (∆ LV GCS) and **d** global radial strain (∆ LV GRS)

We observed significant correlations between improvement in LV GLS with reductions in left ventricular mass index (LVMi) [Pearson’s r = 0.356, p = 0.018]. Improvement in LV GLS paralleled improvements in left ventricular stroke volume index (LVSVi) [Pearson’s r = − 0.429 p = 0.004], left ventricular ejection fraction (LVEF) [Pearson’s r = − 0.408, p = 0.006], LV GCS (Pearson’s r = 0.420, p = 0.004) and LV global radial strain (GRS) [Pearson’s r = − 0.530, p = 0.002] (Fig. 5). There were no significant correlations between LV GLS, T1 or T2 measurements (p > 0.05 for all). Also, we did not find associations between changes in LV GLS with changes in serum levels of creatinine, heart rate or blood pressure (see Table 4). On univariate regression analysis none of the clinical variables examined (age, gender, body mass index (BMI), diabetes, dialysis vintage, type of donor and time after transplantation the first exam was done) were determinants of changes in LV GLS (Table 5).

**Table 4:** Correlation between changes (from baseline to 6 months) in left ventricular systolic strain, CMR and clinical variables

	∆ GLS	∆ GCS	∆ GRS
∆ LV EF	− 0.408 (p = 0.006)	− 0.702 (p < 0.001)	0.667 (p < 0.001)
∆ LV EDVi	− 0.030 (p = 0.845)	0.369 (p = 0.013)	− 0.280 (p = 0.066)
∆ LV ESVi	0.229 (p = 0.134)	0.646 (p < 0.001)	− 0.539 (p < 0.001)
∆ LV SVi∆	− 0.429 (p = 0.004)	− 0.187 (p = 0.224)	0.254 (p = 0.095)
∆ LV Mi	0.356 (p = 0.018)	0.573 (p < 0.001)	− 0.612 (p < 0.001)
∆ Native T1	− 0.008 (p = 0.957)	0.180 (p = 0.242)	− 0.014 (p = 0.930)
∆ T2	− 0.194 (p = 0.206)	0.074 (p = 0.634)	0.080 (p = 0.605)
∆ Creatinine	− 0.045 (p = 0.773)	− 0.208 (p = 0.175)	0.182 (p = 0.236)
∆ HR	0.175 (p = 0.256)	0.046 (p = 0.768)	0.150 (p = 0.330)
∆ SBP	0.012 (p = 0.938)	0.103 (p = 0.502)	− 0.124 (p = 0.423)
∆ DBP	0.109 (p = 0.482)	0.189 (p = 0.218)	− 0.066 (p = 0.671)

*DBP* Diastolic blood pressure, *EDVi* end-diastolic volume index, *EF* ejection fraction, *ESVi* end-systolic volume index, *GCS* global circumferential strain, *GLS* global longitudinal strain, *GRS* global radial strain, *HR* heart rate, *LV* left ventricular, *Mi* mass index, *SBP* systolic blood pressure, *SVi* stroke volume index, ∆ change from baseline to 6 months

**Table 5:** Clinical determinants of ∆ LV GLS by FT-CMR

Independent variable	Dependent variable (∆ LV GLS)		
	B Coef	95% CI	p value
Age at transplantation	− 0.03	(− 0.11; 0.04)	0.350
Gender (female)	− 1.52	(− 3.31; 0.26)	0.092
BMI	0.05	(− 0.17; 0.26)	0.660
Diabetes (No)	− 0.40	(− 2.33; 1.52)	0.673
Dialysis vintage	− 0.01	(− 0.03; 0.01)	0.276
Donor (Deceased)	1.42	(− 0.99; 3.85)	0.241
Time after T*x* 1st exam	0.03	(− 0.24; 0.31)	0.816

*BMI* body mass index, *Tx* transplantation, ∆ change from baseline to 6 months

### Intraobserver reproducibility and validation of LV GLS measurements

**Fig. 6:**
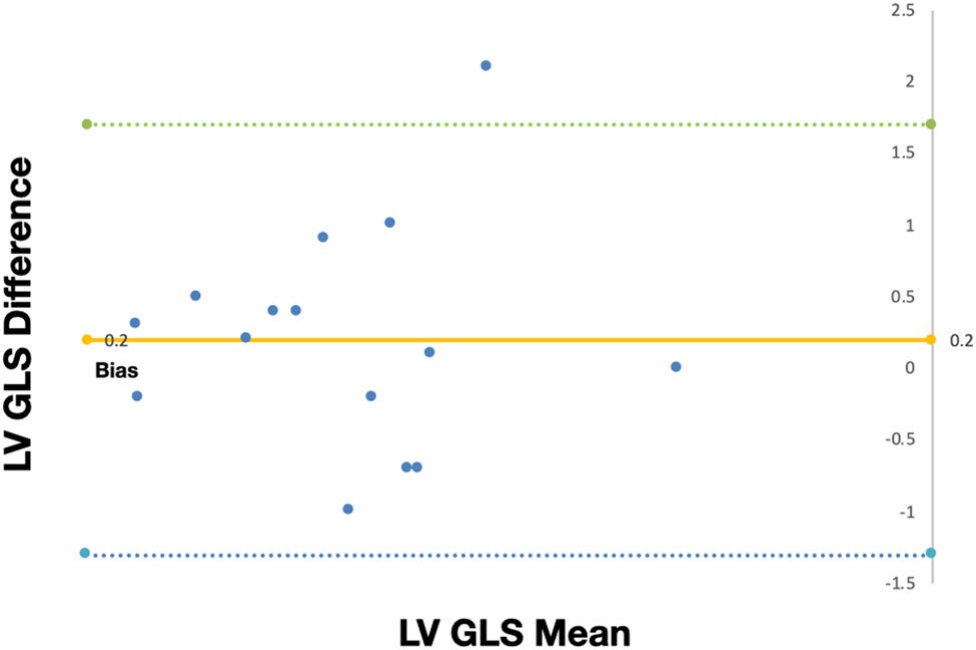
Bland–Altman plot of LV GLS measurements

**Fig. 7:**
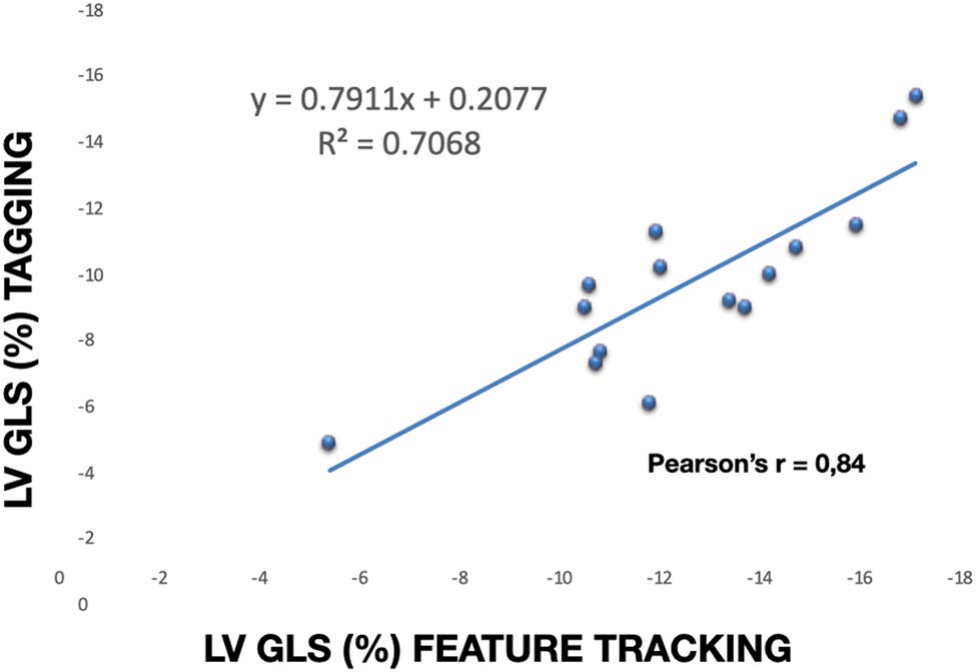
Correlation between LV GLS measured by FT-CMR and tagging

The Bland–Altman analysis for intraobserver reproducibility of LV GLS, measured by FT-CMR, showed a bias of 0.2 and a confidence interval (95% CI) of − 1.3 to 1.7 (Fig. 6). There was a strong positive correlation (r = 0.84) between the LV GLS values measured by FT-CMR and tagging (Fig. 7).

## Discussion

In this prospective study we demonstrated a favorable impact of KT on LV GLS measured by FT-CMR 6 months after surgery, in concordance with previous echocardiographic studies [17, 18]. Although there was an improvement in LV GLS and a reduction of myocardial fibrosis as assessed by T1 mapping after KT, these important prognostic CMR biomarkers did not reach the normal range values characterized in controls, which could help to explain why KTR are still at increased cardiovascular risk. Yet, improvement in LV GLS was associated with reduction of left ventricular hypertrophy (LVH), but not myocardial fibrosis (T1) or edema (T2).

FT-CMR derived LV GLS is a powerful independent predictor of mortality in patients with ischemic or non-ischemic dilated cardiomyopathy [23], reduced [24] and preserved [25] ejection fraction, incremental to common clinical and imaging risk factors. In studies using STE, LV GLS was independently associated with both all-cause and cardiovascular mortality also in CKD, ESRD and KTR [26–28]. To the best of our knowledge, data regarding changes in strain parameters using FT-CMR are limited to the study by Gong et al. [16] who found that LV GCS and GRS, but not GLS, improved 1 year after KT. This apparent discrepancy probably is due to the elapsed time after transplant surgery data were collected, supporting the hypothesis that GLS improves earlier in the course of recovering subclinical myocardial dysfunction. Also, besides no prior studies investigating the temporal sequence of improvement in LV GLS after KT, Enrico et al. [29] showed that LV GLS improved from baseline to 6 months after KT, but remained unchanged in the next 6 months, until 1 year after surgery. Most progressive myocardial diseases predominantly cause subendocardial dysfunction in their early stages, leading to reduction in longitudinal LV mechanics [11]. Transmural involvement results in concomitant subendocardial and subepicardial dysfunction, decreasing myocardial contractility in all directions, with impairment of LV ejection performance [30]. Explanations about why subendocardium is the most vulnerable region include it is the farthest layer from epicardial coronary flow, it undergoes greater variations in pressure and compression in both systole and diastole, and also appears to be more susceptible to early microvascular and structural changes such as fibrosis [31]. So, it is expected that GLS also will recover earlier than other strain parameters after effective cardiac treatment. Yet, different from us, Gong et al. [16] found significant improvement in LVEF 1 year after KT, and that was correlated with improvement in LV GCS and GRS. This finding could be explained because the contribution of midwall circumferential shortening has a greater impact on LV SV and LVEF than longitudinal shortening [32]. In the present study, although we found a significant correlation between improvements in LV GLS with other parameters of systolic function (GCS, GRS and EF), LVEF showed a slight, nonsignificant increase in the follow-up.

Strain measures are susceptible to changes in preload and afterload [33], but in our study, we did not observe significant correlations between LV GLS with end-diastolic volume index (surrogate for preload) or blood pressure (surrogate for afterload), suggesting that improved LV systolic function is likely attributed to other effects of KT, including the removal of uremic toxins, reversal of LVH, and restoration of inflammation and oxidative stress state [34], rather than loading conditions.

Cardiac remodeling, as assessed by LV mass and geometry, is also a strong predictor of cardiovascular and all-cause mortality in studies of asymptomatic populations [35] and in CKD [3]. Contrarily, reverse remodeling after KT is associated with better outcomes in patients with cardiac dysfunction [6]. However, there are discrepancies between STE and CMR exams regarding the effects of successful KT on LVH, a common feature of UC. Similar to Patel et al. [36] we did not observe significant regression in LVMi 6 months after KT, although improvements in LV GLS were associated with reductions in LVMi. Indeed, Stokke et al. [37] demonstrated that LVH can compensate impaired LV GLS for maintaining LVEF, so it is expected that improvement in LV GLS will contribute to reductions in LVH and vice versa, while LVEF remains constant.

LVH may be associated with cardiac fibrosis that leads to conduction disturbances and probably provides the link between UC, arrhythmia and sudden death. The use of newer T1 mapping techniques without gadolinium (native T1) has demonstrated increased myocardial T1 relaxation times indicative of diffuse interstitial fibrosis in ESRD [13, 14] and early-stage CKD [12]. Although KT was associated with reduced septal native T1 [15], probably related to reduction in diffuse interstitial fibrosis, in this study we failed to prove associations between improvements in LV GLS with reductions in native T1. It has been suggested that changes in myocardial systolic and diastolic deformation are functional markers of diffuse interstitial fibrosis. A previous experimental study by Kramann et al. [38] reported that strain parameters not only detected LV contractile abnormalities but also correlated with the severity of interstitial myocardial fibrosis and hypertrophy in rat models with uremic cardiomyopathy. On the other hand, in a recent clinical study, Frojdh et al. [39] demonstrated that myocardial interstitial fibrosis assessed by expansion of extracellular volume fraction (ECV) and GLS were both associated with outcomes, but they correlated minimally, suggesting that diffuse myocardial interstitial expansion and contractile dysfunction may reflect different domains of myocardium disease.

Our study has some limitations. First, the baseline CMR was performed after transplantation surgery. Although performing CMR at the pre-transplant period would be more appropriate, this is not possible with deceased donors due to long waiting list times. However, all patients were clinically and hemodynamically stable at the moment of the CMR exam. Second, we did not perform histological confirmation of myocardial fibrosis because of the inherent risks and decreasing use of endomyocardial biopsy in daily practice, however T1 mapping is a well validated CMR technique to this end. Third, this is a single-center study with a small number of patients, and may have been underpowered to show associations between native T1 and GLS. Fourth, we did not measure serum biomarkers of heart failure, however, previous study has found associations between B‐type natriuretic peptide (BNP) and those CMR indices in hemodialysis patients [40]. Finally, outcome assessment was not feasible, because of the limited follow-up duration.

Overall, our findings reinforce the potential role of multiparametric CMR in monitoring important biomarkers of UC in ESRD patients after KT. Early detection of individuals at higher risk for CVD after KT could allow identification of those who might benefit from closer cardiovascular follow-up or more aggressive therapies. Future studies with increased number of patients and longer follow-up are needed to determine which of these CMR parameters will be helpful in predicting mortality or guiding therapy.

## Conclusion

In this prospective study we demonstrated that LV GLS measured by FT-CMR improves 6 months after KT in association with reverse remodeling but not native T1 or T2 measurements.

## Data Availability

The datasets used and/or analyzed during the current study are available from the corresponding author on reasonable request.

## Abbreviations

BMI: Body mass index
BSA: Body surface area
bSSFP: Balanced steady state free precession
CKD: Chronic kidney disease
CMR: Cardiac magnetic resonance
CVD: Cardiovascular disease
EDV: End-diastolic volume
EF: Ejection fraction
ESRD: End-stage renal disease
ESV: End-systolic volume
FT-CMR: Feature-tracking
GCS: Global circumferential strain
GLS: Global longitudinal strain
GRS: Global radial strain
KT: Kidney transplantation
KTR: Kidney transplant recipients
LV: Left ventricle/ventricular
LVEF: Left ventricular ejection fraction
LVH: Left ventricular hypertrophy
LVM: Left ventricular mass
MOLLI: Modified Look-Locker inversion-recovery
NSF: Nephrogenic systemic fibrosis
RV: Right ventricle/ventricular
STE: Speckle tracking echocardiography
SV: Stroke volume
UC: Uremic cardiomyopathy
